# The PSG challenge: towards comprehensive scene understanding

**DOI:** 10.1093/nsr/nwad126

**Published:** 2023-05-04

**Authors:** Jingkang Yang, Zheng Ma, Qixun Wang, Xiaofeng Guo, Haofan Wang, Ziwei Liu, Wayne Zhang, Xing Xu, Hai Zhang

**Affiliations:** SenseTime Research, China; S-Lab, Nanyang Technological University, Singapore; SenseTime Research, China; Xiaohongshu Inc, China; Institute of Optics and Electronics, Chinese Academy of Sciences, China; Xiaohongshu Inc, China; S-Lab, Nanyang Technological University, Singapore; SenseTime Research, China; School of Computer Science and Engineering, University of Electronic Science and Technology of China, China; Pazhou Laboratory (Huangpu), China; School of Mathematics, Northwest University, China; Pazhou Laboratory (Huangpu), China

## Abstract

The Panoptic Scene Graph Generation (PSG) challenge evaluates computer vision models to identify relations in images beyond object classification and localization, enabling a deeper understanding of scenes for real-world AI applications.

## INTRODUCTION

**Figure 1.**
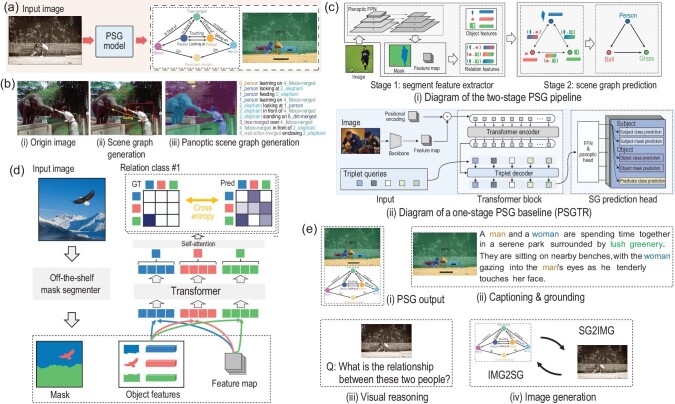
(a) PSG models generate a scene graph to comprehensively describe the input image, with segmentation masks to ground each node (object or background). Adapted from ref. [4]. (b) Difference between (ii) scene graph generation (SGG) and (iii) panoptic scene graph generation (PSG). (c) PSG baselines. Adapted from ref. [4]. (d) Diagram of the winning solution (GRNet). (e) Potential downstream tasks of the PSG model.

Most computer vision tasks currently focus on recognizing objects in isolation. For instance, image classification only needs to identify the main object in an image [1], while object detection and image segmentation only require models to locate objects in images [2]. However, these tasks are insufficient to achieve a comprehensive and in-depth understanding of a scene. For example, in Fig. 1b(i), a model that only detects people, elephants, fences and trees would not have an understanding of the scene, making it unable to provide safety reminders such as not to feed the elephants. In many real-world AI applications like smart cities, autonomous driving and smart manufacturing, it is crucial to not only localize targets in the scene but also reason and predict their relations. For example, in autonomous driving, it is important to analyze whether pedestrians on the side of the road are pushing or riding bicycles. In smart factories, it is necessary to judge whether operators are working correctly. Understanding these relations is vital for decision-making.

Scene graph generation (SGG) goes beyond object classification and localization by predicting relations between objects in a scene [3]. However, traditional scene graph generation has limitations, including inaccurate object localization and limited background annotation, due to limitations in bounding-box annotations (Fig. 1b(ii)) [4]. To overcome these limitations, a new SGG setting named the panoptic scene graph generation (PSG) leverages panoptic segmentation for accurate and comprehensive localization of objects and backgrounds, thus improving the field towards a deeper scene understanding [4]. Formally, as shown in Fig. 1b, the PSG challenge expects the developed PSG models to generate a scene graph with nodes that represent objects or backgrounds, and edges representing the relations between them. PSG models should also accurately segment objects to identify the corresponding nodes in the scene graph.

**Dataset and metrics.** The PSG challenge uses the public PSG dataset [4], which contains 49K images with 133 object/background classes and 56 relation classes. Each image is annotated with panoptic segmentation and scene graphs (see psgdataset.org for dataset details). The evaluation protocol for the PSG challenge consists of two sub-tasks: scene graph detection (or generation), abbreviated SGDet, with the metrics Recall@K and mean Recall@K, and panoptic segmentation with the metric PQ. The main evaluation metric is the (mean) recall rate of the top K triplets that are predicted by the PSG model. For a (Subject, Verb, Object) triplet, a successful recall requires a mask-based IOU over 0.5 for subject and object, and correct classification of all the elements in (Subject, Verb, Object). More details are available in the PSG paper [4].

**Challenges.** We list the following difficulties to be solved by participants.

- *Dealing with relation ambiguity*. Relations can have broad meanings that can apply to various scenarios, such as ‘crossing’ for ‘airplane crossing sky’, ‘car crossing road’ and ‘person crossing road’. The model must learn to understand the meaning of ambiguous relations.
- *Distinguishing similar relations*. The model must differentiate between similar relations, such as ‘running’ and ‘walking’, or ‘parked on’ and ‘driving on’, based on visual cues in the image.
- *Generalizing relations*. The model should be able to generalize relation concepts, even beyond the training set, such as detecting ‘person driving a train’ when trained on ‘person driving a car’.
- *Dealing with partial and imbalanced annotations*. Relations in the PSG dataset are often partially labeled, with many objects, which makes it difficult to annotate all relations. Additionally, the data are also imbalanced, due to the long-tailed nature of the world.

## THE WINNING SOLUTION

The PSG challenge received 100 submissions from teams presenting various solutions. These included utilizing advanced image segmentation methods and addressing long-tail problems. The competition also received several innovative approaches, such as scene graph-specific data augmentation techniques. After careful evaluation based on performance metrics and the novelty and significance of the solutions, GRNet [5] emerged as the winning method. This section will provide an overview of the PSG baselines and delve into the workings of GRNet.

### Preliminary: the PSG baselines

Before introducing the winning solution to the PSG challenge, we first introduce two classic PSG baselines: a two-stage method and a one-stage method [4]. For the two-stage baseline, as shown in Fig. 1c(i), in the first stage, a pretrained panoptic segmentation model, panoptic feature pyramid networks, is used to extract features, masks and class predictions from individual objects in an image. Those individual object features are then fed to a classic scene graph generator such as iterative message passing [3] in the second stage. This two-stage approach allows classic SGG methods to be adapted to the PSG task with minimal modifications. Figure 1c(ii) shows the diagram of a one-stage method PSGTR, which first uses a convolutional neural network to extract the image feature, and then a detection-transformer-like  [6] encoder-decoder is used to learn the triplet representation directly. A Hungarian matcher [7] is used to compare the predicted triplets with ground truth triplets. The optimization objective then maximizes the cost calculated by the matcher, and the total loss is calculated using cross-entropy for labels and DICE/F-1 for segmentation.

### Model architecture

The winning team of the PSG task presented a new method called GRNet [5]. As previous research [4] has shown that one-stage models currently outperform two-stage models, the winning team conjectured that the advantage mainly comes from the direct supervision signals from the image feature map, which is beneficial for capturing relations. However, the winning team also found that one-stage models cannot usually achieve good segmentation performance. Based on this observation, the winning solution aims to find a trade-off between the two paradigms by reviving the two-stage paradigm and equipping it with the ability to obtain global context like the one-stage paradigm.

Specifically, as shown in Fig. 1d, the winning team first adopts an off-the-shelf panoptic segmentation method like Mask2Former [8], which generates masks for each object. The intermediate feature map of a specific object from the segmentor and its corresponding mask are fused as the object-level feature. Instead of handling pairwise objects individually as the classic one-stage paradigm does (shown in Fig. 1c(i)), the winning team proposes building a global context module by utilizing a transformer that processes each object-level content with the feature map that contains global information. Note that a class embedding is also added to indicate the category of the object. With the cross-attention mechanism in the transformer encoder, the output object feature (see Fig. 1d) gathers more global information from other objects. Finally, for each object-level feature, a global average pooling is performed to generate new object embeddings that have been further contextually enriched. A relation-wise binary classification task is performed to determine the existence of relations between object pairs for each relation category.

### Relation classification

The winning team also introduces some special considerations for the relation-wise binary classification task. For example, they note that the PSG dataset often contains two objects having more than one relation, such as ‘person looking at elephant’ and ‘person feeding elephant’ at the same time (see Fig. 1a). To address this, the solution proposed is to transform the relation prediction from a single-label classification problem in their initial attempt to a multi-label classification problem.

Besides, the winning team is also aware of the fact that the PSG dataset strives for precision and relevance in its annotation process by requiring annotators to choose specific and accurate predictions, such as ‘parking on’ instead of more general ones like ‘on’. However, it could be unsuitable for the learning of the boarder relation like ‘on’, since it in fact exists along with ‘parking on’. To resolve the conflict, the winning team proposes a self-training strategy with self-distilled labels for relation classification and uses the exponential moving average to dynamically update the labels.

### Other designs

When computing the loss for relation-wise binary classification, each predicted object must be paired with its corresponding ground truth. The Hungarian matching algorithm is used for this purpose. However, the algorithm is prone to instability, particularly during the early training phase when the network’s accuracy is low. This can lead to different matching outcomes for the same input, causing inconsistent optimization directions for the network and making it harder to train. To address this issue, commonly referred to as ‘matching jitters’, the winning team utilizes denoising training, where noisy real results are fed into the decoder as a shortcut to learn relative offsets, skipping the matching step and allowing for direct learning methods, which effectively overcome the challenge posed by matching jitters.

## AWARD REASONING

The winning solution of the PSG challenge is GRNet, a new method presented by the winning team. The team aims to find a balance between the two-stage paradigm and the one-stage paradigm by reviving the two-stage paradigm and equipping it with the ability to obtain global context like the one-stage paradigm. GRNet first adopts a panoptic segmentation method to generate masks for each object. The intermediate feature map and mask of a specific object are then fused to form an object-level feature. A transformer processes each object-level feature with the global feature map, which is further enriched by the cross-attention mechanism. A global average pooling is performed to generate new object embeddings. Finally, a relation-wise binary classification task is performed to determine the existence of a relation between object pairs for each relation category. The winning team addresses challenges such as the utilization of global information for the two-stage paradigm, the conflict between relation precision and generalization by using a multi-label classification and self-distillation, and the computational efficiency due to the light-weighted two-stage paradigm. More importantly, the winning solution obtains the best overall scores among all participants.

## FUTURE DIRECTIONS

The PSG task refines the problem formulation of scene graph generation and has attracted a large number of researchers to push the development of comprehensive scene understanding models. There are still some interesting problems that need to be addressed in the PSG task, and we hope that future researchers will focus on the following issues.

- *Hierarchical structure analysis of relations*. Relations can generally be divided into location relations and action relations. The algorithms may need to model the hierarchical structure of relations to avoid neglecting a type of relation.
- *Relation recognition through visual reasoning*. A model with additional reasoning ability, even a language model, can be combined with visual reasoning to recognize relations, which is a field that has not yet been fully explored in the field of computer vision.
- *Mutual promotion of relation recognition and image segmentation*. With the main task of relation recognition, we hope that the future PSG model can also improve the image segmentation performance. Intuitively, the current recognition of ‘feeding’ in Fig. 1a might help a more accurate segmentation of the person’s hand. Similarly, relation recognition could also be enhanced if more information is utilized from the accurate masks.

The PSG task opens up new avenues for exciting applications. For instance, in visual captioning tasks [9], the comprehensive information from PSG models can help generate more descriptive captions and prepare for a good visual grounding dataset (Fig. 1e(ii)). In visual reasoning tasks like visual question answering [10], the relation information from PSG models can significantly improve the accuracy of answers (Fig. 1e(iii)). Furthermore, by incorporating PSG models into text-to-image generation techniques like DALLE-2, we can generate images that more accurately reflect the relations described in the text prompt (Fig. 1e(iv)). This can lead to a closed loop where the generated images could be used to further enrich the PSG dataset.

We would like to invite the wider community to explore and imagine the many other applications that can benefit from a model with a good understanding of relations. Beyond visual reasoning, captioning and scene graph-to-image tasks, there are surely a wealth of other applications waiting to be discovered.
